# The Apple Mitogen-Activated Protein Kinase *MdMAPK6* Increases Drought, Salt, and Disease Resistance in Plants

**DOI:** 10.3390/ijms26073245

**Published:** 2025-03-31

**Authors:** Mengru Li, Huaina Gao, Minmin Zhou, Yali Zhang, Han Jiang, Yuanyuan Li

**Affiliations:** State Key Laboratory of Wheat Improvement, Shandong Collaborative Innovation, Center of Fruit & Vegetable Quality and Efficient Production, National Research, Center for Apple Engineering and Technology, College of Horticulture Science and Engineering, Shandong Agricultural University, Taian 271018, China; 2022110296@sdau.edu.cn (M.L.); 17853516338@163.com (H.G.); zmm90302@163.com (M.Z.); ylzhang0522@163.com (Y.Z.)

**Keywords:** apple, *MdMAPK6*, protein kinase, pathogenic bacteria, drought, salt

## Abstract

As sessile organisms, plants are exposed to a variety of environmental stresses caused by biotic and abiotic factors during their life cycle; as a result, plants have evolved complex defense mechanisms to cope with these stresses, among which the mitogen-activated protein kinase cascade signaling pathway is particularly critical. This study focused on *MdMAPK6*, a specific mitogen-activated protein kinase gene in *Malus domestica*, to illuminate its functions in stress responses. *MdMAPK6* was successfully cloned from apple and shown to respond to various stressors, including drought, salt, and abscisic acid. Overexpressing *MdMAPK6* in apple calli resulted in enhanced resistance to drought, salt, and *Botryosphaeria dothidea.* Ectopic expression of *MdMAPK6* in *Arabidopsis thaliana* enhanced the resistance to drought, salt, and *Pseudomonas syringae pathovar tomato* DC3000. These results indicated that *MdMAPK6* in apples is a traditional mitogen-activated protein kinase, which plays an important role in both biotic and abiotic stresses.

## 1. Introduction

As sessile organisms, plants encounter a variety of survival stresses throughout their life cycle [1]. These include abiotic stresses such as drought, salinity, and weather extremes, as well as biotic stresses such as pathogen infection, insect pests, and weed competition [2]. Exposure of plants to these stresses can have serious negative effects on their growth and development, such as reduced yields and overall quality of produce. Therefore, plants have gradually evolved complex adaptive mechanisms, including physiological changes and biochemical responses, to improve their resilience under stress conditions. A thorough understanding of how plants respond to various stresses is essential for improving plant resilience through breeding and biotechnological approaches, as well as for developing sustainable agriculture to mitigate the effects of environmental stresses [3].

External stress signals are first perceived by various types of receptors on plant organs, and subsequently, signaling pathways transmit these molecular signals from the outside of the cell through the plasma membrane to the inside of the cell, enabling the molecular signals inside the cell to act accordingly. This process enables plants to respond quickly and efficiently to changes in the external environment. The mitogen-activated protein kinase (MAPK) cascade is an important pathway for plants responding to adversity stress. The MAPK cascade, a highly conserved signaling module, is ubiquitous in eukaryotes [4]. A typical MAPK cascade consists mainly of MAPK, MAPK kinase (MAPKK), and MAPKK kinase (MAPKKK) [5,6,7]. When plants perceive external stimuli, MAPKKKs are first activated, and phosphorylated MAPKKKs activate directly downstream MAPKKs by phosphorylation of serine and/or threonine residues in the S/T-X_3-5_-S/T motif. The MAPKKs then activate downstream MAPKs by phosphorylating threonine and tyrosine residues in TXY motifs and activated MAPKs can phosphorylate a variety of downstream substrates, including transcription factors, protein kinases and structural proteins, which ultimately triggers a cellular response and enables plants to make adaptive responses to external stimuli [8,9].

The role of MAPKs in plant resistance to external abiotic stresses has been demonstrated in many plants [10,11,12,13]. For example, in *A. thaliana*, point mutations in *AtMAPK9* and *AtMAPK12* lead to increased transpirational water loss from leaves, which reduces plant drought tolerance [14]; in rice, *OsMPKK10.2* positively regulates drought resistance by phosphorylation and activation of two different MAPKs, *OsMAPK6* and *OsMAPK3* [15]; in potato, overexpression of *StMAPK11* can better maintain plant growth and photosynthesis under drought conditions [16]; in tomato, overexpression of *SlMAPK3* can enhance the drought resistance of plants [17]; in cotton, overexpression of *GhMAPK2* can positively regulate the drought and salt resistance of the plant [18]; in maize, overexpression of *ZmMAPK1* enhances drought resistance, and overexpression of *ZmMAPK5* enhances salt resistance in plants [19,20].

Apple ring rot, caused by *Botryosphaeria dothidea*, has emerged as a significant disease in apple production across China, posing a serious threat to the fruit tree industry [21]. This pathogen primarily targets apple branches and fruit tissues. Notably, its infection cycle exhibits distinct overwintering characteristics: *B. dothidea* survives the winter in infested branch tissues, manifesting as mycelium, conidiophores, and ascospores. As spring temperatures rise, the pathogen initiates the primary infection process through two main avenues: first, it directly infects the host tissue using the overwintering mycelium; second, it is spread by conidia carried by rain [22,23,24]. The MAPK signaling cascade pathway plays a crucial regulatory role in plant defense responses to biotic stresses, particularly during pathogen infections. This complex signaling network is essential for how plants perceive and respond to various threats, enabling them to activate their defense mechanisms effectively and enhance their resilience against harmful microorganisms. This cascade of reactions is primarily activated by PAMPs, which are recognized by plant receptors, triggering a series of signaling responses that ultimately lead to the activation of defense mechanisms [25]. For example, in *A. thaliana*, *AtMAPK3* and *AtMAPK6* activated by *B. cinerea* can regulate the biosynthesis of plant phytoalexin by phosphorylating the downstream transcription factor *AtWRKY33* [26]. In addition, they regulate the expression of defense genes by phosphorylating the downstream transcription factor *AtERF6*, thereby enhancing plant resistance to *B. cinerea*. [27]; in tobacco, *NtMEK2* enhances plant resistance to tobacco mosaic virus by phosphorylating *NtSIPK* and *NtWIPK* [28]; in soybean, *GmMPK6*, which is phosphorylated and activated by *GmMKK4*, significantly enhances the expression of defense related genes *GmPR1* and *GmPR10-1* through the phosphorylation of *GmERF113*. This process ultimately increases the resistance of transgenic soybean to *P. sojae* [29]; in cotton, the signaling module *GhMPK9*-*GhRAF39*-*GhWRKY40a* regulates the defense responses mediated by *GhERF1b* and *GhABF2*, thereby initiating plant defense mechanisms against *Verticillium* wilt [30]; in apple, the phosphorylation of *MdMAPKK1* mediated by *MdBSK1* enhances the resistance of apple calli and fruit *to B. dothidea.* [31].

In summary, MAPKs involved in plant resistance to abiotic and biotic stresses were mostly isolated and identified from model crops such as *A. thaliana* and soybean [32,33]. Despite the increasing importance of MAPKs in plant resistance to abiotic and biotic stresses, there is still a relative paucity of relevant reports on MAPKs in apples in these respects. In this study, an MAPK gene named *MdMAPK6* was successfully isolated from an apple, and its biological characteristics and functions were comprehensively and meticulously analyzed to explore the functional properties of *MdMAPK6* in depth. The results showed that when *MdMAPK6* was overexpressed in apple calli or *A. thaliana*, it was able to significantly enhance the drought and salt tolerance, and disease resistance of these plants. This finding not only provides a new research perspective and theoretical basis for the improvement of apple resistance but also provides valuable clues to unravel the operation of the MAPK cascade signaling pathway in response to external abiotic and biotic stresses in apples.

## 2. Results

### 2.1. Bioinformatics Analysis of the MdMAPK6 Gene in Apple

**Figure 1:**
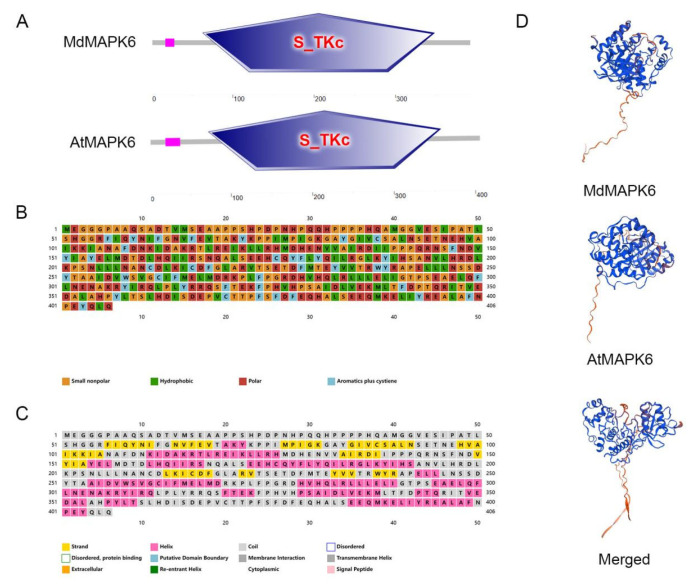
Bioinformatics analysis of MdMAPK6 and AtMAPK6. (**A**) Location of AMP-binding domains in MdMAPK6 and AtMAPK6. (**B**) The amino acid characterization of the MdMAPK6 protein; orange refers to small nonpolar, green refers to hydrophobic, red refers to polar, and blue refers to aromatics plus cysteine. (**C**) The prediction of the MdMAPK6 protein’s secondary structure; yellow refers to strands, pink refers to helices, and gray refers to coils. (**D**) The prediction of the tertiary structure of the MdMAPK6 protein, AtMAPK6 protein, and their overlap.

**Figure 2:**
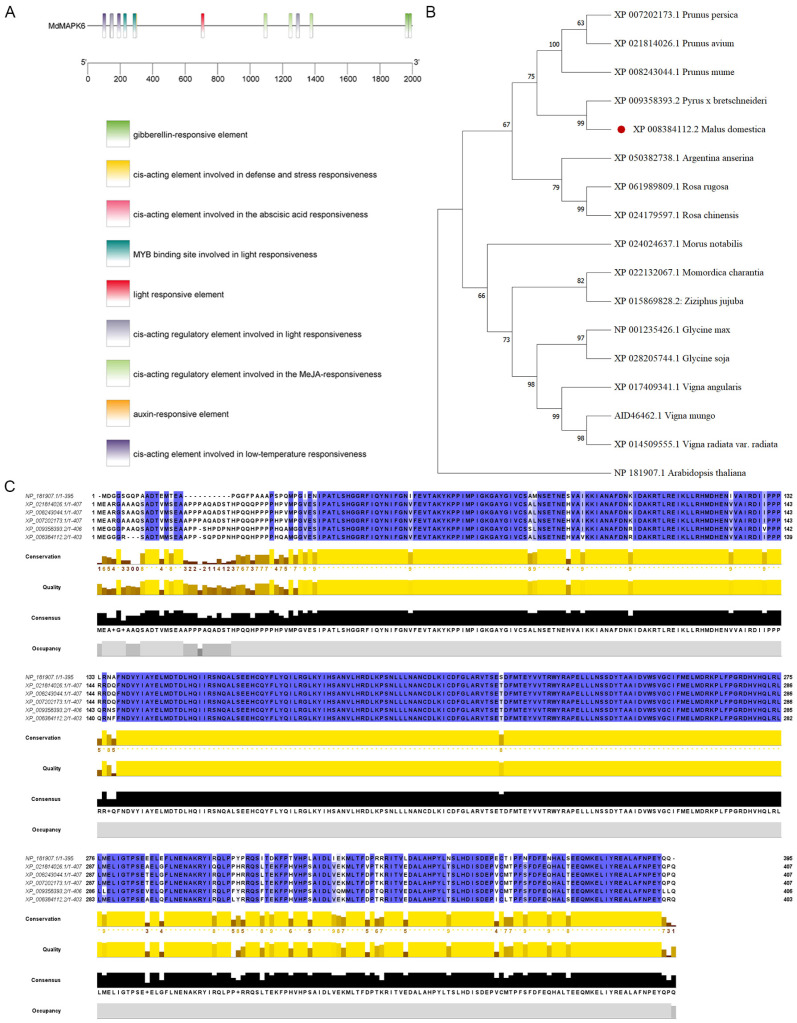
Cis-acting element analysis of the *MdMAPK6* promoter region, phylogenetic tree, and amino acid multiple sequence alignment of the MAPK6 protein. (**A**) The promoter components were analyzed by PlantCARE and then visualized using TBtools-II (Toolbox for Biologists) v2.067, which included nine different cis-acting elements. (**B**) The phylogenetic tree constructed from MAPK6 proteins of 17 different species: *P. persica*, *P. avium*, *P. mume*, *P. x bretschneideri*, *M. domestica*, *A. anserina*, *R. rugosa*, *R. chinensis*, *M. notabilis*, *M. charantia*, *Z. jujuba*, *G. max*, *G. soja*, *V. angularis*, *V. mungo*, *V. radiata var*. *radiata*, and *A. thaliana*. (**C**) Multiple sequence alignment of the MAPK6 protein from different species: NP_181907.1 (*A. thaliana*), XP_021814026.1 (*P. avium*), XP_008243044.1 (*P. mume*), XP_007202173.1 (*P. persica*), XP_009358393.2 (*P. x bretschneideri*), and XP_008384112.2(*M. domestica*).

After searching in the apple genome database, we identified the homolog of *A. thaliana AtMAPK6* (AT2G43790) in the apple as MD15G1147300 and named it *MdMAPK6* as the target gene for the subsequent study (Supplementary Figure S1). Firstly, we carried out a bioinformatics analysis. We learned that the coding sequence of *MdMAPK6* is 1221 base pairs long, the protein generated from this coding sequence contains 406 amino acids, and the predicted molecular weight of this protein is 46.28 kDa. The protein structural domains of MdMAPK6 and AtMAPK6 were analyzed by using the SMART online tool, and the results showed that both of them contain, at the same or similar positions, a serine/threonine kinase (S-TKc) structural domain at the same or similar position (Figure 1A). The amino acids are mainly composed of four categories: small nonpolar, hydrophobic, polar, and aromatics plus cysteine (Figure 1B). The secondary structure is mainly composed of four different conformational forms: alpha helices with a share of 41.87%, random coil structures with a share of 38.92%, extended strands with a share of 13.05%, and beta turns with a share of 6.16% (Figure 1C and Supplementary Figure S2). The prediction results of the tertiary structure of the MdMAPK6 protein and AtMAPK6 protein obtained through the SWISS-MODEL website showed that the tertiary structure of the two proteins was highly similar, indicating that they may have similar functions (Figure 1D). According to the analysis of cis-acting elements on the *MdMAPK6* promoter predicted by the PlantCARE website, it mainly includes defense, stress, low temperature, gibberellic acid, abscisic acid, jasmonic acid, auxin, light, and other relevant response elements (Figure 2A). To explore the relationship between MdMAPK6 and MAPK6 of other species, we found the protein sequences of MAPK6 of 16 other species on the NCBI website and constructed a phylogenetic tree through MEGA-64 software; the results showed that MAPK6 in *Pyrus x bretschneideri* was the closest relative to MAPK6 in *M. domestica* (Figure 2B, Supplementary Table S3). The multiple sequence alignment on MAPK6 of *A. thaliana*, *Prunus avium*, *Prunus mume*, *Prunus persica*, *P. x bretschneideri,* and *M. domestica* showed that MAPK6 has high protein sequence homology among species, indicating that the MAPK6 protein is evolutionarily conserved and plays critical functional roles across species (Figure 2C).

### 2.2. Expression Profiles of MdMAPK6 in Different Apple Tissues

**Figure 3:**
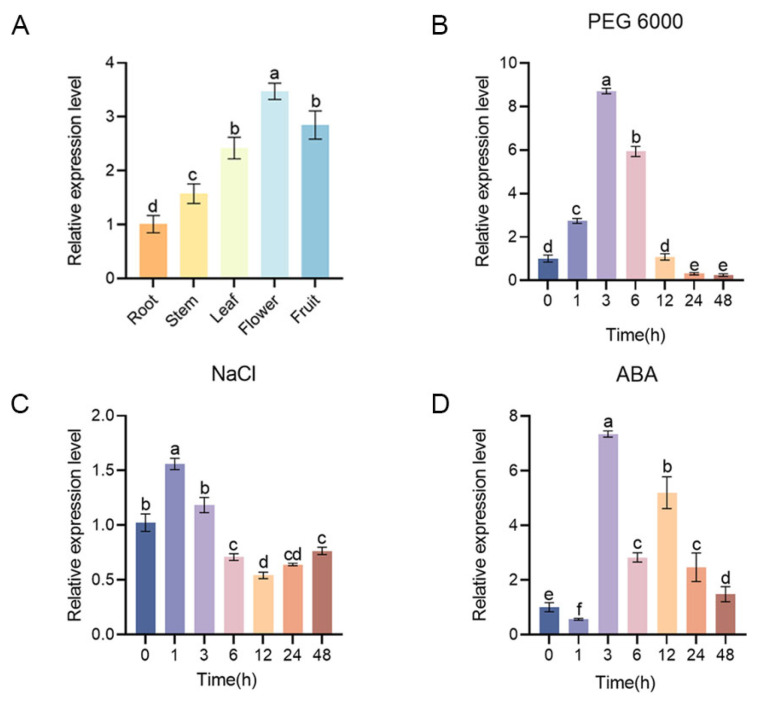
The relative expression analysis of *MdMAPK6*. (**A**) The relative expression levels of *MdMAPK6* in the roots, stems, leaves, flowers, and fruits of apple detected by qRT-PCR. The relative expression levels of *MdMAPK6* after (**B**) PEG 6000, (**C**) NaCl, and (**D**) ABA detected by qRT-PCR. Data were obtained from three independent biological replicates. Different lowercase letter markers represent significant differences, and the significance level is taken as 0.05.

To study the specific expression of *MdMAPK6* in different tissues of apple, we detected the expression levels of *MdMAPK6* in the roots, stems, leaves, flowers, and fruits of ’Royal Gala’ apple by qRT-PCR, and the results showed that *MdMAPK6* was expressed in all the above five kinds of apple tissues, with higher expression in the leaves, flowers, and fruits of apple (Figure 3A).

### 2.3. Responses of MdMAPK6 to PEG 6000, NaCl, and ABA Treatments

To verify the response of *MdMAPK6* to abiotic stresses, we subjected apple seedlings to PEG 6000, NaCl, and ABA treatments and subsequently analyzed *MdMAPK6* expression. Our results showed a transient increase in the expression of *MdMAPK6* under all three treatments (Figure 3B–D). Notably, the expression of *MdMAPK6* showed a biphasic rise after ABA treatment (Figure 3D). In conclusion, *MdMAPK6* may be involved in plant response to abiotic stress.

### 2.4. Overexpression of MdMAPK6 Enhances Drought Salt Tolerance While Decreasing the Sensitivity of Apple Calli to ABA

**Figure 4:**
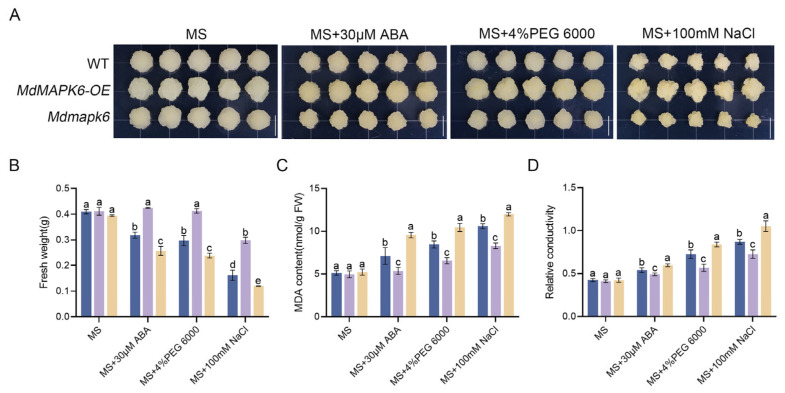
Phenotypes of the WT and *MdMAPK6* transgenic lines under different abiotic stress conditions. (**A**) Phenotypes of the WT and *MdMAPK6* lines treated with MS, MS + 30 µM ABA, MS + 4% PEG 6000, and MS + 100 mM NaCl. Bars = 1 cm. (**B**) Fresh weight, (**C**) MDA content, and (**D**) relative conductivity of the WT and *MdMAPK6* transgenic lines’ apple calli after treatments. FW stands for fresh weight. Data were obtained from three independent biological replicates. The different lowercase letter markers represent significant differences, and the significance level is taken as 0.05.

To investigate the biological function of *MdMAPK6* in apple calli in response to PEG 6000, NaCl, and ABA treatments, we successfully cloned the *MdMAPK6* gene from the cDNA template of the ‘Royal Gala’ apple. Subsequently, using an *Agrobacterium*-mediated genetic transformation system, we created an *MdMAPK6* overexpression line (*MdMAPK6-OE*) and a corresponding silencing line (*Mdmapk6*) driven by the 35S promoter in apple calli. The expression level of *MdMAPK6* in *MdMAPK6-OE* is 5.77 times that of the WT, while in *Mdmapk6*, it is only 0.27 times that of the WT (Supplementary Figure S3). We selected a 15-day-old wild-type apple calli as a control and treated it with the following four media conditions: MS medium, MS medium with 30 µM ABA, MS medium with 4% PEG 6000 to simulate drought stress, and MS medium with 100 mM NaCl to simulate salt stress. Concurrently, the aforementioned stress treatments were applied to the wild-type calli and to *MdMAPK6*-*OE* and *Mdmapk6* transgenic lines, to conduct a detailed analysis of the specific mechanism of action of *MdMAPK6* under these stress conditions. The results showed that all three treatments significantly inhibited the growth of WT calli. In contrast, the *MdMAPK6-OE* line exhibited growth restriction only under NaCl stress, yet its biomass accumulation remained significantly higher than that of the WT. Notably, the *Mdmapk6* mutant displayed a more pronounced growth inhibition phenotype across all three treatment conditions (Figure 4A). By analyzing the effect of treatments, we found that the fresh weight of WT calli was lower in all treatments than in the absence of any stimuli. This loss of weight caused by the different stresses was more dramatic for *Mdmapk6* calli, while the fresh weight of *MdMAPK6-OE* calli was like that of the control, except for those under NaCl stress (Figure 4B). Subsequently, the MDA content and relative conductivity were also analyzed. The experimental results showed that *MdMAPK6-OE* exhibited significantly lower MDA content and relative conductivity after stress treatment compared to the WT, whereas *Mdmapk6* showed the opposite response pattern, with significantly higher MDA content and relative conductivity than the WT (Figure 4C,D). MDA, a byproduct of membrane lipid peroxidation, serves as a critical marker for the extent of oxidative damage inflicted upon cellular membranes, while the relative conductivity reflects the integrity and permeability of cell membranes. Altogether, these results revealed that *MdMAPK6* enhances the tolerance to all tested treatments by negatively regulating oxidative stress and cell death.

### 2.5. Overexpression of MdMAPK6 Enhances Drought and Salt Tolerance and Decreases the Sensitivity of A. thaliana to ABA

**Figure 5:**
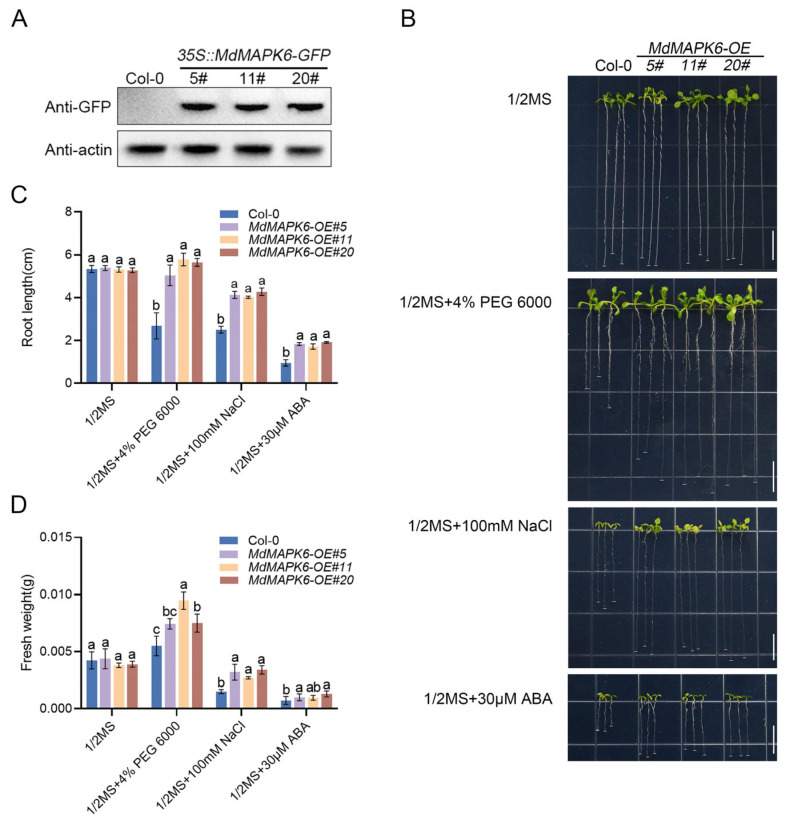
Phenotypes of Col-0 and *MdMAPK6* transgenic lines of *A. thaliana* seedlings under different abiotic stress conditions. (**A**) MdMAPK6-GFP protein abundance in different *35S::MdMAPK6-GFP* lines, as determined by an immunoblot with an anti-GFP antibody. (**B**) Phenotypes of Col-0 and *MdMAPK6* transgenic lines of *A. thaliana* seedlings treated with 1/2 MS, 1/2 MS + 4% PEG 6000, 1/2 MS + 100 mM NaCl, and 1/2 MS + 30 µM ABA. Bars = 1 cm. (**C**) Root length and (**D**) fresh weight of Col-0 and *MdMAPK6* transgenic lines of *A. thaliana* seedlings. Data were obtained from three independent biological replicates. The different lowercase letter markers represent significant differences, and the significance level is taken as 0.05.

To further investigate the mechanism of *MdMAPK6* in enhancing drought and salt tolerance in plants, we employed *Agrobacterium*-mediated genetic transformation to successfully construct overexpression transgenic lines carrying GFP tags in the model plant *A. thaliana.* On this basis, we screened three overexpression transgenic lines, *MdMAPK6-OE#5*, *MdMAPK6-OE#11*, and *MdMAPK6-OE#20,* for subsequent detailed experimental studies (Figure 5A and Supplementary Figure S4). We used four different medium conditions, including standard 1/2 MS medium, 1/2 MS medium containing 4% PEG 6000, 1/2 MS medium containing 100 mM NaCl, and 1/2 MS medium containing 30 µM ABA, for Col-0 and *MdMAPK6*-overexpressing transgenic lines. The experimental results showed that these three lines did not exhibit significant differences in growth conditions compared to the WT. However, after treatment, the three overexpression transgenic lines grew significantly better than the Col-0, and showed stronger stress tolerance (Figure 5B). Then, we quantified the root length and fresh weight of Col-0 and overexpression transgenic lines. The results showed that the root length of the overexpression transgenic lines was significantly increased compared to Col-0 (Figure 5C), and the fresh weight was also obviously increased (Figure 5D). In conclusion, the overexpression of *MdMAPK6* enhances tolerance to drought and salinity and decreases sensitivity to ABA in *A. thaliana* as observed in apple calli.

### 2.6. Overexpression of MdMAPK6 Enhances Resistance of Apple Calli to B. dothidea

**Figure 6:**
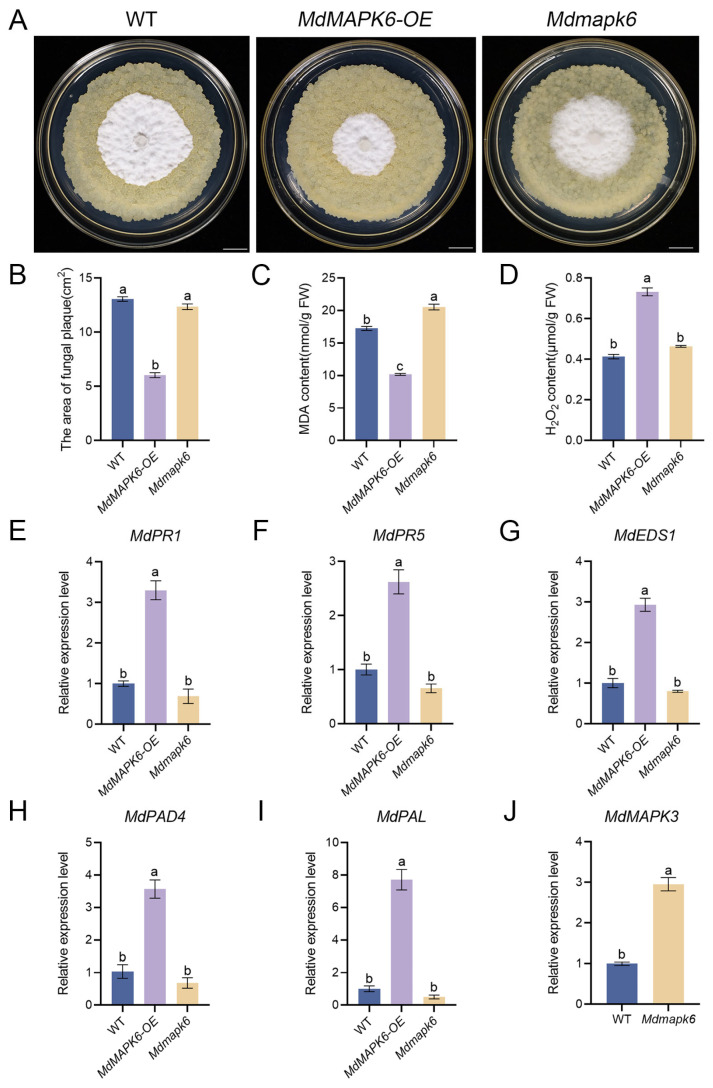
Overexpression of *MdMAPK6* enhances the resistance of apple calli to *B. dothidea.* (**A**) Phenotypes of WT apple calli and *MdMAPK6* transgenic lines after inoculation with *B. dothidea* for 7 days. Bars = 1 cm. (**B**) The area of fungal plaque, (**C**) MDA content, and (**D**) H_2_O_2_ content of WT apple calli and *MdMAPK6* transgenic lines after infection with *B. dothidea* for 7 days. (**E**–**I**) Relative expression of disease-resistant genes of WT apple calli and *MdMAPK6* transgenic lines 7 days after infection with *B. dothidea* detected by qRT-PCR. (**J**) Relative expression levels of *MdMAPK3* in WT apple calli and *MdMAPK6*-silencing lines detected by qRT-PCR. Data were obtained from three independent biological replicates. The different lowercase letter markers represent significant differences, and the significance level is taken as 0.05.

To determine whether *MdMAPK6* is involved in apple calli defense against *B. dothidea*, we inoculated a 15-day-old WT, an *MdMAPK6* overexpression line (*MdMAPK6*-*OE*), and an *MdMAPK6*-silencing line (*Mdmapk6*) with *B. dothidea* and observed the growth of *B. dothidea*. The radial elongation diameter of mycelia was used as one of the indexes to evaluate apple calli resistance. After 7 days of inoculation, *MdMAPK6*-*OE* showed significantly lower mycelial diameter and mycelial growth rate compared to the WT, whereas *Mdmapk6* showed no significant differences in mycelial diameter and growth compared to the WT (Figure 6A,B). According to the detection of MDA content in apple calli after inoculation, the MDA content in apple calli of *MdMAPK6*-*OE* was significantly reduced compared with the WT, while the MDA content in apple calli of *Mdmapk6* was significantly increased compared with the WT (Figure 6C). In the process of disease resistance, the accumulation of H_2_O_2_ will not only cause plant defense reactions to trigger plant immunity but also cause direct damage to pathogenic organisms. Therefore, we also tested the H_2_O_2_ content and found that the H_2_O_2_ content in the apple calli of *MdMAPK6*-*OE* was significantly increased, while the H_2_O_2_ content in the apple calli of *Mdmapk6* had no significant change compared with the WT (Figure 6D). In addition, we further detected the expression levels of the salicylic acid-responsive genes *MdPR1*, *MdPR5*, *MdEDS1*, *MdPAD4,* and *MdPAL* by qRT-PCR in the apple calli of WT, *MdMAPK6*-*OE*, and *Mdmapk6* after fungal inoculation. The results showed that the expressions of all tested genes in the apple calli of *MdMAPK6*-*OE* were significantly higher than those in WT, contrary to the expression in the apple calli of *Mdmapk6* (Figure 6E–I), indicating that *MdMAPK6* enhances the resistance of apple calli to *B. dothidea*.

Given that the susceptibility of *Mdmapk6* to *B. dothidea* was similar to infected WT calli, we speculated the presence of redundant *MdMAPK6* activity in apple calli. Previously, Zipeng Yu et al. (2023) showed redundant activity between *AtMAPK3* and *AtMAPK6* [34]. Therefore, we studied the expression of *MdMAPK3* in the WT and *Mdmapk6* apple calli after inoculation with *B. dothidea* by qRT-PCR. The results showed that the expression level of *MdMAPK3* in the apple calli of *Mdmapk6* was significantly higher than that in the WT (Figure 6J), supporting the hypothesis.

### 2.7. Overexpression of MdMAPK6 Enhances the Resistance of Apple Fruit to B. dothidea

**Figure 7:**
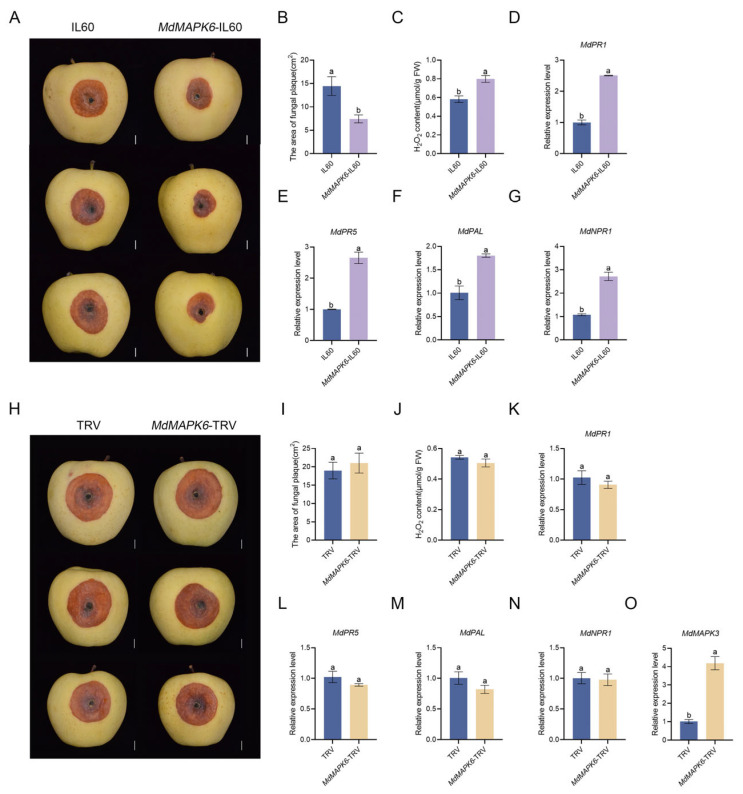
Overexpression of *MdMAPK6* enhances the resistance of apple fruit to *B. dothidea.* (**A**) Phenotypes of IL60 and *MdMAPK6*-IL60 in apple fruit 7 days after inoculation with *B. dothidea*. Bars = 1 cm. (**B**) The area of fungal plaque and (**C**) H_2_O_2_ content in apple fruit of IL60 and *MdMAPK6*-IL60 after inoculation with *B. dothidea* for 7 days. (**D**–**G**) Relative expression of disease-resistant genes in apple fruit of IL60 and *MdMAPK6*-IL60 after inoculation with *B. dothidea* for 7 days detected by qRT-PCR. (**H**) Phenotypes of TRV and *MdMAPK6*-TRV in apple fruit 7 days after inoculation with *B. dothidea*. Bars = 1 cm. (**I**) The area of fungal plaque and (**J**) H_2_O_2_ content in apple fruit of TRV and *MdMAPK6*-TRV after inoculation with *B. dothidea* for 7 days. (**K**–**N**) Relative expression of disease-resistant genes in apple fruit of TRV and *MdMAPK6*-TRV after inoculation with *B. dothidea* for 7 days detected by qRT-PCR. (**O**) The relative expression of *MdMAPK3* in apple fruit of TRV and *MdMAPK6*-TRV after inoculation with *B. dothidea* for 7 days detected by qRT-PCR. Data were obtained from three independent biological replicates. The different lowercase letter markers represent significant differences, and the significance level is taken as 0.05.

We also performed transient transfection of apple fruit and inoculation experiments with *B. dothidea*. In this study, we utilized the IL-60 viral plasmid vector to achieve overexpression of *MdMAPK6* in apple fruit, while applying the TRV viral vector to achieve silencing of *MdMAPK6* in apple fruit. The expression of *MdMAPK6* in IL60, *MdMAPK6*-IL60, TRV, and *MdMAPK6*-TRV was quantitatively detected after apple fruit was placed in darkness for 3 days after transient transfection. The results showed that *MdMAPK6*-IL60 significantly elevated the expression of *MdMAPK6* compared with IL60. Meanwhile, *MdMAPK6*-TRV significantly decreased the expression of *MdMAPK6* compared with TRV (Supplementary Figure S5). The phenotypes were observed 7 days after the inoculation of *B. dothidea.* The plaque necrosis area of *MdMAPK6*-IL60 apple fruit was significantly smaller than that of IL60 (Figure 7A,B). The detection results of H_2_O_2_ content in apple fruit of IL60 and *MdMAPK6*-IL60 showed that the H_2_O_2_ content of *MdMAPK6*-IL60 was significantly higher than that of IL60 (Figure 7C). We also quantified the expression of the disease-resistant genes *MdPR1*, *MdPR5*, *MdPAL,* and *MdNPR1*, and showed that the expression of all the genes evaluated was significantly higher in *MdMAPK6*-IL60 compared with IL60 (Figure 7D–G). The above results indicate that the overexpression of *MdMAPK6* enhances apple fruit resistance to *B. dothidea*. However, we found that the phenotypes of TRV and *MdMAPK6*-TRV did not change significantly 7 days after inoculation (Figure 7H). The results of the quantification of the plaque necrotic area (Figure 7I), H_2_O_2_ content (Figure 7J), and expression levels of the disease-resistant genes *MdPR1*, *MdPR5*, *MdPAL*, and *MdNPR1* (Figure 7K–N) were all consistent with the phenotypes. The results were the same as those of the inoculation of the silencing lines of apple calli. Subsequently, we quantitatively detected the expression of *MdMAPK3* in TRV and *MdMAPK6*-TRV apple fruit after inoculation for 7 days, and the results showed that the expression of *MdMAPK3* in *MdMAPK6*-TRV was significantly higher compared with TRV (Figure 7O). These results provide new evidence for our conjecture that *MdMAPK3* and *MdMAPK6* are a pair of redundant genes in the process of apple resistance against *B. dothidea*.

### 2.8. Overexpression of MdMAPK6 Enhances Resistance to Pst DC3000 in A. thaliana

**Figure 8:**
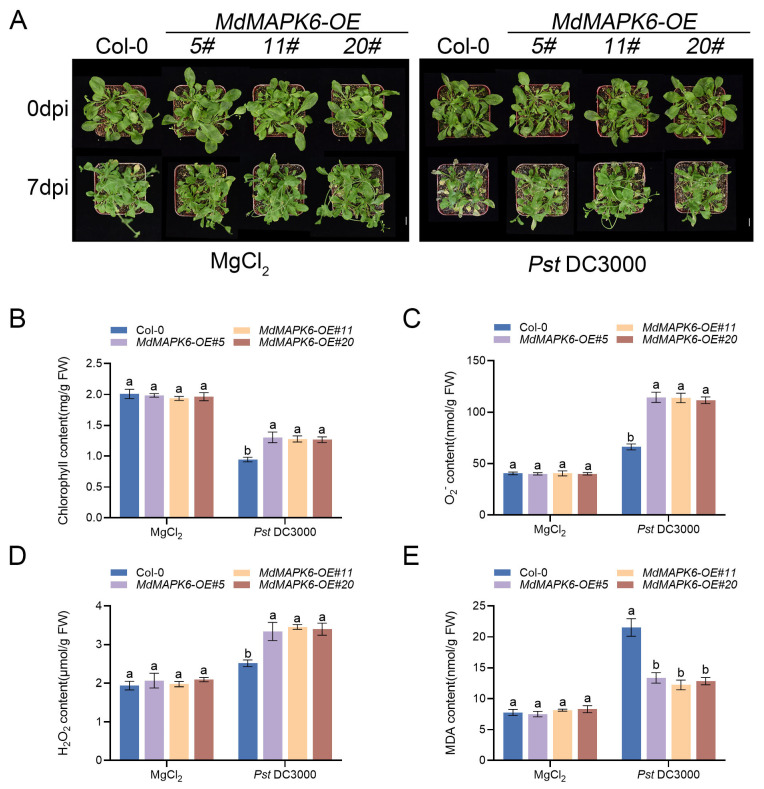
Overexpression of *MdMAPK6* enhances the resistance of *A. thaliana* to *Pst* DC3000. (**A**) Phenotypes of Col-0 and *MdMAPK6* transgenic lines of *A. thaliana* after inoculation with *Pst* DC3000 for 3 h (0 dpi) and 7 days (7 dpi). (**B**) Chlorophyll content, (**C**) O_2_^−^ content, (**D**) H_2_O_2_ content, and (**E**) MDA content of Col-0 and *MdMAPK6* transgenic lines *A. thaliana* after inoculation with *Pst* DC3000 for 7 dpi. Bars = 1 cm. Data were obtained from three independent biological replicates. The different lowercase letter markers represent significant differences, and the significance level is taken as 0.05.

In order to further explore the role of *MdMAPK6* in plant resistance to pathogenic bacteria, we selected *Pst* DC3000 as the model pathogen and systematically compared the phenotypic differences in disease resistance between Col-0 and three *MdMAPK6*-overexpressing lines (*MdMAPK6-OE#5*, *MdMAPK6-OE#11*, and *MdMAPK6-OE#20*) in the pathogen inoculation experiment. At the same time, an equal dose of 10 mM MgCl_2_ was injected as a control. It was later observed that there was no significant difference in growth between Col-0 and the three *MdMAPK6* overexpression lines at 0 dpi and 7 dpi in the control group. After inoculation with 0 dpi, there was no significant difference in the growth of Col-0 and the three *MdMAPK6* overexpression lines. With time, the leaves of Col-0 showed obvious yellowing, drying, and necrosis than before at 7 dpi. The three *MdMAPK6* overexpression lines showed only slight leaf yellowing at 7 dpi, showing milder disease than Col-0 (Figure 8A). Subsequently, the chlorophyll, MDA, H_2_O_2_, and O_2_^−^ contents of Col-0 and the three *MdMAPK6* overexpression lines were detected at 7 dpi. The results showed that there was no significant difference in chlorophyll, MDA, H_2_O_2_, and O_2_^−^ content between Col-0 and the three *MdMAPK6* overexpression lines in the control group. After inoculation with *Pst* DC3000, the chlorophyll, O_2_^−^, and H_2_O_2_ contents of the three *MdMAPK6* overexpression lines were significantly higher than those of Col-0 (Figure 8B–D), while the MDA content was significantly lower than that of Col-0 (Figure 8E). These results indicate that overexpression of *MdMAPK6* can enhance the resistance of *A. thaliana* to *Pst* DC3000.

### 2.9. MdMAPKK4 and MdMAPKK5 Interact with MdMAPK6

**Figure 9:**
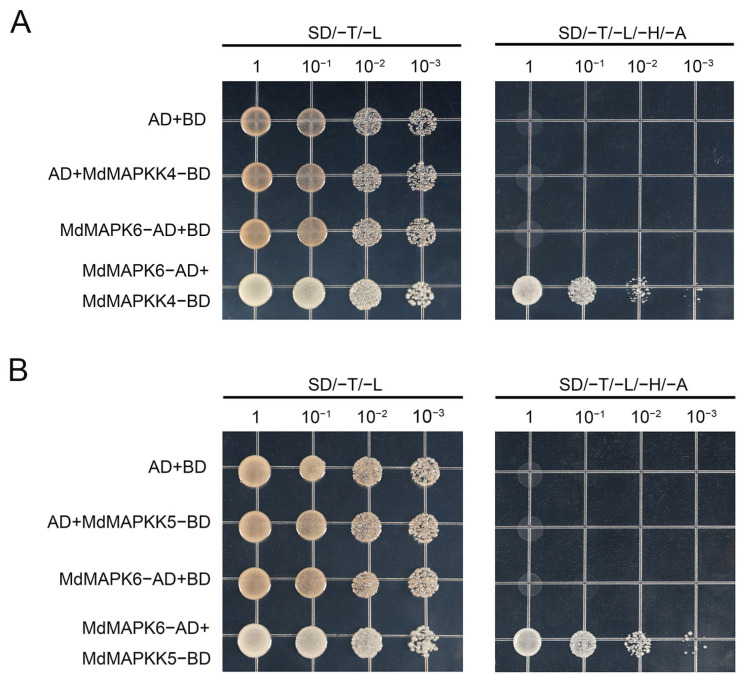
MdMAPKK4 and MdMAPKK5 interact with MdMAPK6. (**A**) A Y2H assay demonstrated the interaction of MdMAPKK4 and MdMAPK6 in the yeast system. (**B**) A Y2H assay demonstrated the interaction of MdMAPKK5 and MdMAPK6 in the yeast system.

Previous studies have shown that *AtMAPKK4* and *AtMAPKK5* are upstream MAPKKs of *AtMAPK6* in *A. thaliana.* To explore whether the interaction between MAPKK4/MAPKK5 and MAPK6 still exists in apple, we first constructed recombinant plasmids MdMKK4-BD, MdMKK5-BD, and MdMAPK6-AD, and then verified it by a yeast two-hybrid (Y2H) experiment. The results show that MdMAPKK4 and MdMAPKK5 interact with MdMAPK6 in apples (Figure 9A,B).

## 3. Discussion

In plants, the MAPK cascade signaling module exhibits a high degree of conservation, a property that confers its ability to efficiently transmit extracellular signals to the cell, thus enabling the cell to rapidly sense and respond to external stimuli, and then to take adaptive measures in response to environmental changes [35]. It has been shown that the MAPK cascade pathway, through its modular structure, plays a central regulatory role in a number of biological processes, among which are the response mechanisms to biotic and abiotic stresses [36].

It has been shown that *GmMAPK6* is involved in the drought stress response of soybean seedlings through a positive regulatory mechanism [37]. In *Arabidopsis*, it was reported that transgenic *A. thaliana* plants overexpressing *MnMAPK1* showed significantly increased root length compared with the wild type after 10 days of treatment with 250 mM NaCl, confirming its function in enhancing plant salt tolerance [38]. This study reveals the universal drought resistance function of *MdMAPK6* across species and developmental stages using apple calli, Arabidopsis seedlings, and mature Arabidopsis plants. The experimental data showed that both MDA content and the relative conductivity of *MdMAPK6* overexpression lines were significantly reduced compared with the WT, suggesting that *MdMAPK6* may enhance plant tolerance to drought and salt by regulating redox homeostasis and cell membrane stability.

The study conducted by Jinggeng Zhou et al. (2022) offers valuable insights into the mechanisms through which the MAPK pathway enhances plant defense against pathogenic invaders. The results showed that in *A. thaliana*, the increased susceptibility to *B. cinerea* in *Atmapk3* mutants compared to Col-0 suggests that *AtMAPK3* can enhance plant resistance to *B. cinerea* [39]. In this study, we revealed that *MdMAPK6* enhances plant resistance to bacteria or fungi through apple calli, apple fruits, and *A. thaliana*. The experimental data showed that *MdMAPK6* overexpression lines triggered a specific H_2_O_2_ burst accumulation under *B. dothidea* infection, a phenomenon coupled with the regulatory mechanism of MAPK-ROS signaling in plant immunity [40,41]. We also observed that MDA content in *MdMAPK6-OE* did not accumulate despite the ROS burst. We hypothesize that this phenomenon may be attributed to the plant’s simultaneous enhancement of antioxidant enzyme activities and non-enzymatic antioxidant substances upon pathogen infection, which efficiently scavenged excessive ROS and thereby blocked lipid peroxidation chain reactions [42]. Further investigations demonstrated systematic activation of key regulatory nodes (*MdEDS1*, *MdPAD4*, etc.) in the SA biosynthesis pathway within *MdMAPK6* overexpression lines, which aligns with the canonical MAPK-SA signaling axis in disease resistance [43,44,45,46]. These findings collectively indicate that *MdMAPK6* likely participates in pathogen defense through dual regulatory mechanisms: establishing an oxidative defense barrier by precisely modulating ROS homeostasis, while simultaneously activating core components of the SA signaling pathway to implement systemic resistance. Our pathogen infection assays in apple calli and transient expression systems demonstrated that the *MdMAPK6*-silencing line exhibited susceptibility to *B. dothidea* that was comparable to the WT. Because the functional redundancy of MAPK3 and MAPK6 in coordinating pathogen defense in different plant species has been well documented [47,48,49], we hypothesized that this compensatory mechanism might be evolutionarily conserved in apple. To test this hypothesis, a comparative analysis of *MdMAPK3* expression was conducted between the WT and the *MdMAPK6*-silencing line at 7 days post-*B. dothidea* inoculation. Quantitative analysis revealed significant compensatory upregulation of *MdMAPK3* transcript levels in the silencing line, providing preliminary evidence for functional redundancy between *MdMAPK3* and *MdMAPK6* in mediating apple’s resistance to pathogenic bacterial infection.

Emerging evidence from model plant systems *(A. thaliana, O. sativa, etc.)* has established that MAPKK4/5-MAPK6 phosphorylation partnerships constitute evolutionarily conserved signaling modules [50,51,52,53,54]. Our study extends this paradigm to apple through systematic validation using in planta protein–protein interaction assays. Yeast two-hybrid analysis conclusively demonstrated the formation of canonical phosphorylation relays between MdMAPKK4/MdMAPKK5 and MdMAPK6, confirming the phylogenetic preservation of this kinase cascade architecture in apple.

**Figure 10:**
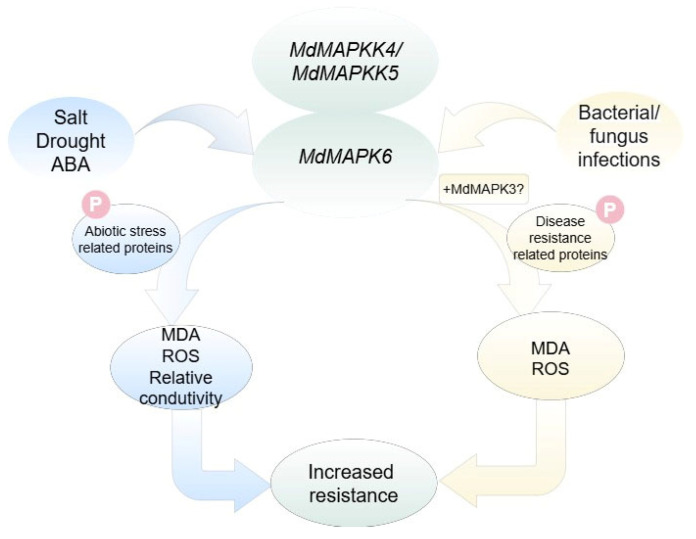
A model for the involvement of MdMAPK6 in drought, salt, ABA, and pathogen stress in plants. In response to drought, salt, and ABA stress, *MdMAPK6* may activate abiotic stress-related genes by phosphorylation, reducing MDA and H_2_O_2_ content and relative conductance to attenuate cellular oxidative damage, and modulating the ROS signaling pathway, thereby enhancing plant tolerance to drought, salt, and ABA stress. During bacterial/fungal infection, *MdMAPK6* may work together with *MdMAPK3* to activate disease resistance-related genes in the SA biosynthesis pathway by coordinating ROS homeostasis and phosphorylation, thereby significantly upregulating the expression of these genes and enhancing plant disease resistance. And *MdMAPK6* interacts with *MdMAPKK4* and *MdMAPKK5*.

In summary, *MdMAPK6* in apple is a typical MAPK protein family member. In response to drought, salt stress, and ABA stress, *MdMAPK6* may activate the expression of abiotic stress-related genes through phosphorylation of specific transcription factors, which could reduce lipid peroxidation and cell death, thereby enhancing plant tolerance to drought, salt, and ABA stress. During bacterial/fungal infection, *MdMAPK6* may work together with *MdMAPK3* to activate disease resistance-related genes in the SA biosynthesis pathway by coordinating ROS homeostasis and phosphorylation, thereby significantly upregulating the expression of these genes and enhancing plant disease resistance. These findings highlight the central role of *MdMAPK6* in the regulatory network of plant resistance and provide important molecular targets for breeding more resistant apple varieties. In addition, the interaction of MdMAPK6 with MdMAPKK4 and MdMAPKK5 revealed its evolutionary conservation in apples with other species (Figure 10).

## 4. Materials and Methods

### 4.1. Plant Materials and Growth Conditions

The experimental materials were obtained from the Royal Gala apple trees at the Experimental Station of Shandong Agricultural University, which were used to detect the expression of the *MdMAPK6* gene in apple roots, stems, leaves, flowers, and fruits.

Apple group seedlings of Royal Gala were grown on Murashige and Skoog (MS) medium containing sucrose, 6-benzylaminopurine, Indole-3-acetic acid, Gibberellin A3, and agar. They were incubated for 30 days in an incubation room at 23 °C, 60% relative humidity, and a photoperiod of 16 h light/8 h darkness. Apple seedlings of Royal Gala were treated with 10% polyethylene glycol 6000 (PEG 6000), 100 mmol·L^−1^ NaCl, and 100 µmol·L^−1^ abscisic acid (ABA) for 0 h, 1 h, 3 h, 6 h, 12 h, 24 h, and 48 h [55,56].

Wild-type apple calli (Orin) and *MdMAPK6* transgenic lines (*MdMAPK6*-OE, *Mdmapk6*) were cultured on MS medium containing sucrose, 6-benzylaminopurine, 2, 4-dichlorophenoxyacetic acid, and agar for 15 days at 23 °C under dark conditions. The “Orin” apple calli used in this experiment were obtained from the original material kept in our laboratory.

*A. thaliana* seedlings, including Col-0 and ectopic expression transgenic lines of *MdMAPK6* (*MdMAPK6*-OE#5, *MdMAPK6*-OE#11, *MdMAPK6*-OE#20), were grown on 1/2 MS medium containing sucrose and agar. *A. thaliana* seeds were vernalized and incubated in a light incubator with a photoperiod of 16 h of light/8 h of darkness, 60% relative humidity, and a room temperature of 23 °C.

### 4.2. Bioinformatics Analysis of the MdMAPK6

The basic information about the *MdMAPK6* gene mainly comes from the apple genome (https://iris.angers.inra.fr/gddh13/) accessed on 11 February 2023 for example, the number of base pairs and the number of amino acids. The protein-conserved domains of *MdMAPK6* and *AtMAPK6* were identified using SMART (http://smart.embl-heidelberg.de/) accessed on 8 March 2023. The amino acid properties of *MdMAPK6* are derived from PSIPRED (http://bioinf.cs.ucl.ac.uk/psipred/) accessed on 8 March 2023. *MdMAPK6* amino acid secondary structure information was obtained from PSIPRED (http://bioinf.cs.ucl.ac.uk/psipred/) accessed on 8 March 2023 and SOPMA (https://npsa.lyon.inserm.fr/cgi-bin/secpred_sopma.pl) accessed on 8 March 2023. The protein tertiary structure of *MdMAPK6* and *AtMAPK6* and their comparative analysis were visualized by SWISS-MODEL (https://swissmodel.expasy.org/) accessed on 8 March 2023.

### 4.3. Cis-Acting Element Analysis, Phylogenetic Tree, and Multiple Sequence Alignment

The cis-acting elements in the *MdMAPK6* promoter (2000 bp upstream of the transcription start site) were identified using the PlantCARE (https://bioinformatics.psb.ugent.be/webtools/plantcare/html/) accessed on 9 March 2023website and visualized using TBtools-II (Toolbox for Biologists) v2.067. The National Center for Biotechnology Information (https://www.ncbi.nlm.nih.gov/) accessed on 9 March 2023 identified 17 different MAPK6 proteins, and then the phylogenetic tree was constructed and visualized with MEGA-64 software. The protein sequences required for conserved structural domain comparison were obtained from the National Center for Biotechnology Information (https://www.ncbi.nlm.nih.gov/) accessed on 9 March 2023, and then Jalview 2.11.4.1 software was used to visualize the conserved structural domain images.

### 4.4. Construction of MdMAPK6 Expression Vector and Genetic Transformation of Transgenic Materials

To construct the *MdMAPK6* overexpression vector and antisense vector, we first cloned the full-length cDNA sequence of *MdMAPK6* from tissue culture seedlings of Gala apples by using specific amplification primers and connected it to the pRI-101 plant expression vector [57]. The specific primers are shown in Supplementary Table S1. The *MdMAPK6* overexpression vector and antisense vector were transferred into Agrobacterium. *MdMAPK6* transgenic material was obtained using Agrobacterium-mediated methods.

### 4.5. Plant Total RNA Extraction and Quantitative Real-Time PCR (qRT-PCR) Analysis

Total RNA from the root, stem, leaf, flower, and fruit tissues of apple, apple tissue culture seedling leaves, apple calli, and *A. thaliana* leaves was extracted using an OminiPlant RNA Kit (CWBIO, Beijing, China). RNA reverse transcription to cDNA was completed using a HiScript II 1st Strand cDNA Synthesis Kit (Vazyme, Nanjing, China). The quantitative real-time polymerase chain reaction was used for analysis using an UltraSYBR Mixture (Low ROX) (CWBIO, Beijing, China) and the 2^−ΔΔCT^ calculation method. *Md18S* rRNA was used as an internal control. Specific information on the fluorescence quantitative primers is shown in Supplementary Table S2.

### 4.6. DNA Extraction

The leaves of Col-0 and ectopic expression transgenic lines of *MdMAPK6* were ground with liquid nitrogen and extracted by a plant genomic DNA extraction kit (TIANGEN, Beijing, China).

### 4.7. Protein Extraction

The leaves of Col-0 and ectopic expression transgenic lines of *MdMAPK6* were ground with liquid nitrogen. Then, an appropriate amount of protein extract was added (including 1 M Tris-HCl, 0.5 M Na_2_EDTA, 1% PVPK30, β-mercaptoethanol, and sucrose) and the mixture was incubated on ice for 20 min. Finally, it was centrifuged for 15 min, and the supernatant was retained.

### 4.8. Western Blot

The protein extract was separated on 10% SDS-PAGE gel and then transferred to polyvinylidene difluoride membranes using an electrotransfer device. The membrane was incubated successively in the sealing solution, the primary diluent, and the secondary diluent. The membrane was then immersed in a Western blot developer and developed using a protein imaging device.

### 4.9. The 30 µM ABA, 4% PEG 6000, and 100 mM NaCl Treatments in Apple Calli and A. thaliana

After growing the WT apple calli and the *MdMAPK6* transgenic lines on MS medium for 15 days, they were clumped into spherules of similar sizes using tweezers. They were transferred to MS medium, MS + 30 µM ABA, MS + 4% PEG 6000, and MS + 100 mM NaCl for 15 days to observe the phenotype. And the relevant physiological indexes were detected.

*A. thaliana* seeds were sterilized with 90% ddH_2_O and 10% NaClO, then spread on 1/2 MS medium, vernalized at 4 °C for 3 days, and placed in an incubator at 23 °C for germination. After 3 days, selected Col-0 and *MdMAPK6* transgenic *A. thaliana* plants with a similar growth status were transferred to 1/2 MS medium, 1/2 MS + 4% PEG 6000, 1/2 MS + 100 mM NaCl, and 1/2 MS + 30 µM ABA for 14 days to observe the phenotype. The root length was measured using Digimizer 4.3.4.0 software, and the fresh weight was measured using a 1/10,000 balance.

### 4.10. The Apple Calli and A. thaliana Pathogen Inoculation

The apple calli were grown on the MS medium for 15 days and then flattened onto the new MS medium for treatment. After 3 days, *B. dothidea* cultured on potato dextrose agar (PDA) medium was inoculated into the center of apple calli and placed in an incubator at 28 °C. The phenotype was observed 7 days after inoculation. And the relevant physiological indexes were detected. Then, the expression of physiological indexes and genes related to the course of disease was detected.

The *A. thaliana* was transplanted into the substrate after germination and continued to grow for about 30 days before inoculation with *Pst* DC3000. *Pst* DC3000 was cultured in LB liquid medium at 28 °C until OD_600_ = 1. Finally, *Pst* DC3000 was diluted to OD_600_ = 0.1 with 10 mM MgCl_2_, and the dorsal side of *A. thaliana* rosette leaves was inoculated with a 1 mL needle. Meanwhile, *A. thaliana* with the same number of seeds was selected, and the same dose of 10 mM MgCl_2_ was injected from the back of the rosette leaves of *A. thaliana* as the control [58].

### 4.11. Transient Transfection of Apple Fruit and Inoculation of B. dothidea

First, *MdMAPK6*-IL60 was constructed, the auxiliary plasmid and the plasmid of *MdMAPK6*-IL60 were diluted to 5–10 ng·uL^−1^ with 10 mM MgCl_2_, the diluted auxiliary plasmid and the plasmid of *MdMAPK6*-IL60 were mixed in a certain proportion, and quantitative injection was carried out on apple fruits with a 1 mL syringe. At the same time, the same amount of IL60 plasmid mixture was injected into the other side of the same apple using the same process as *MdMAPK6*-IL60. The *MdMAPK6*-TRV was constructed, the auxiliary plasmid and the plasmid of *MdMAPK6*-TRV were transformed into Agrobacterium, and the Agrobacterium was diluted to OD_600_ = 0.4–0.6 with 10 mM MgCl_2_. The diluted bacterial solution was mixed in a certain proportion, and quantitative injection was carried out on apple fruits with a 1 mL syringe. At the same time, on the other side of the same apple, an equal amount of TRV bacterial liquid mixture treated with the same steps as *MdMAPK6*-TRV was injected. The injected apple fruit was inoculated with *B. dothidea* after 3 days of dark treatment. The apple fruit was placed in an environment with a temperature of 28 °C and a humidity of 80%.

### 4.12. Relevant Physiological Indicator Detection

Malondialdehyde (MDA) content: We weighed about 0.1 g of plant material into a mortar using a 1/10,000 balance, added 2 mL of phosphate buffer to grind it into a homogenate, transferred it to a test tube, and added 5 mL of 2-thiobarbituric acid. It was heated for 10–15 min in boiling water. After the test tube was cooled, the absorbance at 450 nm, 532 nm, and 600 nm was measured by an ultraviolet spectrophotometer with 2-thiobarbituric acid as the blank. The calculation formula is as follows: MDA content = [6.452 × (OD_532_−OD_600_)−0.59 × OD_450_] × Vt/(Vs × W), where ‘Vt’ represents the total volume of the extracted liquid, ‘Vs’ represents the volume of the measured extracted liquid, and ‘W’ represents the fresh weight of the sample.

Relative conductivity: We weighed about 0.1 g of plant material into a mortar using a 1/10,000 balance, ground it with a mortar, transferred it to a test tube, and added 10 mL ddH_2_O; after standing for 2 h, the initial conductance value (S1) was detected with a conductance meter; after testing, the sample was placed in a boiling water bath for 30 min; then, the final conductance (S2) was measured after the sample was cooled to room temperature. The relative conductivity is S1/S2.

Chlorophyll content: We weighed about 0.1 g of leaves from the plant to be tested using a 1/10,000 balance, then added anhydrous ethanol to it and soaked it in the dark at room temperature overnight. The absorbance at 649 nm, 655 nm, and 665 nm was measured by an ultraviolet spectrophotometer with anhydrous ethanol as the blank. The calculation formula is as follows: Chlorophyll content = [(13.95 OD_655_−6.88 OD_649_) × Vt/W] + [(24.96 OD_649_−7.32 OD_665_) × Vt/W], where ‘Vt’ represents the total volume of the extracted liquid and ‘W’ represents the fresh weight of the sample.

H_2_O_2_ content was detected using a H_2_O_2_ content kit (COMINBIO, Suzhou, China); O_2_^−^ content was detected using an O_2_^−^ content kit (COMINBIO, Suzhou, China).

### 4.13. Yeast Two-Hybrid (Y2H) Assays

Recombinant plasmids were constructed by inserting the open reading frame of *MdMAPK6*, *MdMMAPKK4,* and *MdMAPKK5* into pGAD424 and pGBT9 vectors. The appropriate combinations of these recombinant plasmids were cotransformed into Saccharomyces cerevisiae strain Y2H, plated on medium without Trp and Leu (SD/−Trp/−Leu), and incubated for 2 days at 28 °C. To detect interactions, the yeast cells were diluted to 1, 10^−1^,10^−2^, and 10^−3^ with ddH_2_O, and then transformants were plated on a medium without Trp, Leu, His, and Ade (SD/−Trp/−Leu/−His/−Ade) and incubated for 2 days at 28 °C [59]. The specific primers are shown in Supplementary Table S1.

### 4.14. Data Presentation and Statistical Analysis

Three replicates were set for each experiment, and the data were the analysis results of three parallel experiments. The significant differences in all data were analyzed by the LSD method in the DPS data processing system. The same lowercase letter markers represent non-significant differences, different lowercase letter markers represent significant differences, and the significance level is taken as 0.05.

## 5. Conclusions

*MdMAPK3* and *MdMAPK6* exhibit significant functional redundancy in response to pathogen infection in plants. Further studies revealed that MdMAPK6 interacts with the upstream kinases MdMAPKK4 and MdMAPKK5, which reinforces its central position in the signaling network. Particularly strikingly, *MdMAPK6* not only effectively promoted plant resistance to pathogen infection but also significantly enhanced plant tolerance to salt and drought stress, a finding that broadened our understanding of the multifunctionality of *MdMAPK6* in plant adversity responses.

## Data Availability

All data presented in this study can be found in the article or in the Supplementary Materials.

## Supplementary Materials

The following supporting information can be downloaded at: https://www.mdpi.com/article/10.3390/ijms26073245/s1.

## Abbreviations

The following abbreviations are used in this manuscript:

MAPK	mitogen-activated protein kinase
MAPKK	mitogen-activated protein kinase kinase
MAPKKK	mitogen-activated protein kinase kinase kinase
*B. dothidea*	Botryosphaeria dothidea
*Pst* DC3000	Pseudomonas syringae pv. tomato DC3000
PAMPs	pathogen-associated molecular patterns
Col-0	Columbia-0
WT	wild type
MS	Murashige and Skoog
PEG 6000	polyethylene glycol 6000
ABA	abscisic acid
PDA	potato dextrose agar
MDA	malondialdehyde
FW	fresh weight
PAL	phenylalanine ammonia lyase
PR1	pathogenesis-related 1
PR5	pathogenesis-related 5
EDS1	enhanced disease susceptibility 1
PAD4	peptidylarginine deiminase 4
qRT-PCR	quantitative reverse transcription polymerase chain reaction
Y2H	yeast two-hybrid
