# Supplementary material for: The Apple Mitogen-Activated Protein Kinase MdMAPK6 Increases Drought, Salt, and Disease Resistance in Plants
Source: Int J Mol Sci. 2025 Mar 31;26(7):3245. doi: 10.3390/ijms26073245 (PMC11989477; doi:10.3390/ijms26073245)
Supplement: Supplementary file 1 [file ijms-26-03245-s001.zip › ijms-3524112-supplementary.pdf]

Supplementary Material

Supplementary table S1. Specific sequence of cloning primers.

MdMAPK6-F	ATGGAGGGAGGAGGGCCCGCAGCTCAAT
MdMAPK6-R	CTGTAGCTGATACTCGGGGTTAAATGCA
MdMAPK6-PRI-F	TTGATACATATGCCCGTCGACATGGAGGGAGGAGGGCCCGCA
MdMAPK6-PRI-R	TTTACCCATGAATTCGGATCCCTGTAGCTGATACTCGGGGTTA
MdMAPK6-ANTI-F	TTGATACATATGCCCGTCGACCACGTGATCTCTGCCAGGAA
MdMAPK6-ANTI-R	TTTACCCATGAATTCGGATCCAAAATATGTGATTTTGGACT
MdMAPK6-IL60-F	TCTGAATTCGTCGACAAGCTTATGGAGGGAGGAGGGCCCGCAGCTCAAT
MdMAPK6-IL60-R	CCCCCACACGTGTGGTCTAGACTGTAGCTGATACTCGGGGTTAAATGCA
MdMAPK6-TRV-F	AGAAGGCCTCCATGGGGATCCCACGTGATCTCTGCCAGGAA
MdMAPK6-TRV-R	TGTCTTCGGGACATGCCCGGGAAAATATGTGATTTTGGACT
MdMAPK6-AD-F	AAAGAGATCGAATTCCCGGGGATGGAGGGAGGAGGGCCCGCAGCTCAAT
MdMAPK6-AD-R	CTACGATTCATAGATCTCTGCAGGCTGTAGCTGATACTCGGGGTTAAATGCA
MdMAPKK4-BD-F	ACTGTATCGCCGGAATTCCCGGGGATGAGGCCGATTCAATCGCC
MdMAPKK4-BD-R	GCCCGGAATTAGCTTGGCTGCAGGAGAAGAAAGAGGCCTCGGTGG
MdMAPKK5-BD-F	ACTGTATCGCCGGAATTCCCGGGGATGAGGCCGATGCAGCCG
MdMAPKK5-BD-R	GCCCGGAATTAGCTTGGCTGCAGGAGAAGAAAGAGGCCTCGGTGGAG

Supplementary table S2. Specific sequences of quantitative primers

Primer	Sequence
MdMAPK6-F	AGAAGTTTCCTCACGTCCATCC
MdMAPK6-R	TGAGCTAGGGCATCTTCAACAG
MdMAPK3-F	TGAACTCTTCAGACTACACTGCC
MdMAPK3-R	TCCCAAGAAGCTCTGTCAATAGG
MdPR1 -F	CGATTGCAGTGAAGATGTGGGTTT
MdPR1-R	CCATCAAACCCAACACAATGTCCTTA
MdPR5-F	CAAGTGAAAGCGGCTGATGGGAG
MdPR5-R	GTCCACCACTGCAGGTAAATGTGC
MdEDS1-F	GAAACTGAATGGGCAACGATC
MdEDS1-R	CATAAGGCTCGGTACTCTTCG
MdPAD4-F	GGAGTTTGTAAAACTGGGAAGCATTTAC
MdPAD4-R	CATAAGGCTCGGTACTCTTCG
MdPAL-F	CCTCAAGATTCTGCGAGAAGGATCTC
MdPAL-R	GCTCAACAAGCACTTGCCTCAGTT
MdNPR1-F	GATCTTACGCTGGATGGGAGGAAAG
MdNPR1-R	GAAAGCGAGGCTTCTCCAAGCAAC
18S-F	ACACGGGGAGGTAGTGACAA
18S-R	CCTCCAATGGATCCTCGTTA

Supplementary table S3. Phylogenetic tree of *MdMAPK6* specific protein sequences.

Gene	Protein Sequence
XP_007202173.1 [Prunus persica]	MEARGAAAQSADTVMSEAAPPPAQADSTHPQQQPPPPHPVMPGV ESIPATLSHGGRFIQYNIFGNVFEVTAKYKPPIMPIGKGAYGIVCSA LNSETNEHVAIKKIANAFDNKIDAKRTLREIKLLRHMDHENVVAIR DIIPPPRRDQFNDVYIAYELMDTDLHQIIRSNQALSEEHCQYFLYQI LRGLKYIHSANVLHRDLKPSNLLLNANCDLKICDFGLARVTSETD FMTEYVVTRWYRAPELLLNSSDYTAAIDVWSVGCIFMELMDRKP LFPGRDHVHQLRLLMELIGTPSETELGFLNENAKRYIRQLPPHRRQ SLTEKFPHVHPSAIDLVEKMLTFDPTKRITVEDALAHPYLTSLHDIS DEPVCMTPFSFDFEQHALTEEQMKELIYREALAF
XP_021814026.1 [Prunus avium]	MEARGAAAQSADTVMSEAAPPPAQADSTHPQQQPPPPHPVMPGV ESIPATLSHGGRFIQYNIFGNVFEVTAKYKPPIMPIGKGAYGIVCSA LNSETNEHVAIKKIANAFDNKIDAKRTLREIKLLRHMDHENVVAIR DIIPPPRRDQFNDVYIAYELMDTDLHQIIRSNQALSEEHCQYFLYQI LRGLKYIHSANVLHRDLKPSNLLLNANCDLKICDFGLARVTSETD FMTEYVVTRWYRAPELLLNSSDYTAAIDVWSVGCIFMELMDRKP LFPGRDHVHQLRLLMELIGTPSEAELGFLNENAKRYIQQLPPHRR QSLTEKFPHVHPSAIDLVEKMLTFVPTKRITVEDALAHPYLTSLHDI SDEPVCMTPFSFDFEQHALTEEQMKELIYREALAFNPEYQPQ
XP_008243044.1	MEARGSAAQSADTVMSEAAPPPAQADSTHPQQHPPPPHPVMPGV

[Prunus mume]	ESIPATLSHGGRFIQYNIFGNVFEVTAKYKPPIMPIGKGAYGIVCSA LNSETNEHVAIKKIANAFDNKIDAKRTLREIKLLRHMDHENVVAIR DIIPPPRRDQFNDVYIAYELMDTDLHQIIRSNQALSEEHCQYFLYQI LRGLKYIHSANVLHRDLKPSNLLLNANCDLKICDFGLARVTSETD FMTEYVVTRWYRAPELLLNSSDYTAAIDVWSVGCIFMELMDRKP LFPGRDHVHQLRLLMELIGTPSETELGFLNENAKRYIRQLPPHRRQ SLTEKFPHVHPSAIDLVEKMLTFDPTKRITVEDALAHPYLTSLHDIS DEPVCMTPFSFDFEQHALTEEQMKELIYREALAFNPEYQPQ
XP_009358393.2 [Pyrus x bretschneideri]	MEGGGPAAQSADTVMSEAAPPSHPDPNHPQQHPPPPHQAMGGVE SIPATLSHGGRFIQYNIFGNVFEVTAKYKPPIMPIGKGAYGIVCSSL NSETNEHVAIKKIANAFDNRIDAKRTLREIKLLRHMDHENVVAIRD IVPPPQRNSFNDVYIAYELMDTDLHQIIRSNQALSEEHCQYFLYQIL RGLKYIHSANVLHRDLKPSNLLLNANCDLKICDFGLARVTSETDF MTEYVVTRWYRAPELLLNSSDYTAAIDVWSVGCIFMELMDRKPL FPGRDHVHQLRLLLELIGTPSEVELQFLNENAKRYIRQLPFYRRQS FTEKFPHVHPSAIDLVQMMLTFDPTQRITVEDALAHPYLTSLHDIS DEPVCMTPFSFDFEQHALTEEQMKELIYREALAFNPEYLLQ
XP_050382738.1 [Argentina anserina]	AAPSGDAEMSEAGPTQQSAPPHQMGAADAIPAKLSHGGRFIQYNI FGNVFEVTAKYKPPIMPIGKGAYGIVCSALNSETSEHVAIKKIANA FDNKIDAKRTLREIKLLRHMDHENVVAIRDIIPPPLRNVFNDVYIAY ELMDTDLHQIIRSNQALSEEHCQYFLYQILRGLKYIHSANVLHRDL KPSNLLLNANCDLKICDFGLARVTSETDFMTEYVVTRWYRAPELL LNSSDYTAAIDVWSVGCIFMELMDRKPLFPGRDHVHQLRLLMELI GTPSEAELGFLNENAKRYIRQLPLYRRQSFTEKFPQVHPSAIDLVE KMLTFDPTKRITVEDALAHPYLTSLHDISDEPVCMTPFSFDFEQHA LSEEQMKELIYREALAFNPEYQ
XP_061989809.1 [Rosa rugosa]	QSADTVMSEAGPTQQPPAPHQHQMGAEAIPATLSHGGRFIQYNIF GNIFEVTAKYKPPIMPIGKGAYGIVCSALNSDTSEHVAIKKIANAFD NKIDAKRTLREIKLLRHMDHENVVAIRDIIPPPQRTVFNDVYIAYEL MDTDLHQIIRSNQALSEEHCQYFLYQILRGLKYIHSANVLHRDLKP SNLLLNANCDLKICDFGLARVTSETDFMTEYVVTRWYRAPELLLN SSDYTAAIDVWSVGCIFMELMDRKPLFPGRDHVHQLRLLMELIGT PSEAELGFLNENAKRYIRQLPLYRRQSFTEKFPQVHPSAIDLVEKM LTFDPTKRITVEDALAHPYLTSLHDISDEPVCTTPFSFDFEQHALSE EQMKELIYREALAFNPEYQ
XP_024179597.1 [Rosa chinensis]	AQSADTVMSEAGPTQQPPAPHHQMGAEAIPATLSHGGRFIQYNIF GNIFEVTAKYKPPIMPIGKGAYGIVCSALNSDTSEHVAIKKIANAFD NKIDAKRTLREIKLLRHMDHENVVAIRDIIPPPQRTVFNDVYIAYEL MDTDLHQIIRSNQALSEEHCQYFLYQILRGLKYIHSANVLHRDLKP SNLLLNANCDLKICDFGLARVTSETDFMTEYVVTRWYRAPELLLN SSDYTAAIDVWSVGCIFMELMDRKPLFPGRDHVHQLRLLMELIGT PSEAELGFLNENAKRYIRQLPLYRRQSFTEKFPQVHPSAIDLVEKM LTFDPTKRITVEDALAHPYLTSLHDISDEPVCMTPFSFDFEQHALSE EQMKELIYREALAFNPEYQ

XP_024024637.1<br>[Morus notabilis]	MEGAGAAQPSDTVMSEAAPPQGEHQNPPPPQQHIGMENIPATLSH<br>GGRFIQYNIFGNIFEVTAKYKPPIMPIGKGAYGIVCSALNSETNEHV<br>AIKKIANAFDNKIDAKRTLREIKLLRHMDHENVVAIRDIIPPPQRET<br>FNDVYIAYELMDTDLHQIIRSNQALSEEHCQYFLYQILRGLKYIHS<br>ANVLHRDLKPSNLLLNANCDLKICDFGLARVTSETDFMTEYVVTR<br>WYRAPELLLNSSDYTAAIDVWSVGCIFMELMDRKPLFPGRDHVH<br>QLRLLMELIGTPSEAELGFLNENAKRYIRQLQLYRRQSFTEKFPHV<br>HPLAIDLVEKMLTFDPRQRITVEDALAHPYLTSLHDISDEPVSLSPF<br>IFDFEQHALTEEQMKELIYREALAFNPEYQ
XP_022132067.1<br>[Momordica<br>charantia]	MDDGGAAQPDDTVMSEAASVPPQQHDPAAHQHQPPPMGMENIP<br>ATLSHGGRFIQYNIFGNIFEVTAKYKPPIMPIGKGAYGIVCSALNSE<br>TNEHVAIKKIANAFDNKIDAKRTLREIKLLRHMDHENVVAIRDIIPP<br>PQRATFNDVYIAYELMDTDLHQIIRSNQALSEEHCQYFLYQILRGL<br>KYIHSANVLHRDLKPSNLLLNANCDLKICDFGLARVTSETDFMTE<br>YVVTRWYRAPELLLNSSDYTAAIDVWSVGCIFMELMDRKPLFPG<br>RDHVHQLRLLLELIGTPSEADLGFLNENAKRYIRQLPIYQRQSFTE<br>KFPHVHPAAIDLVEKMLTFDPGRRITVEDALAHPYLTSLHDISDEP<br>VCMTPFSFDFEQHALTEDQMKELIYREALAFNPEYHHQ
XP_015869828.2<br>[Ziziphus jujuba]	MDGGTAQRPDTVMSEASAAPPPASDPNHQQHQQVGMDNIPATLS<br>HGGRFIQYNIFGNIFEVTAKYKPPIMPIGKGAYGIVCSALNSETNEH<br>VALKKIANAFDNKIDAKRTLREIKLLRHMDHENVVAIRDIIPPPQR<br>ETFNDVYIAYELMDTDLHQIIRSNQALSEEHCQYFLYQILRGLKYI<br>HSANVLHRDLKPSNLLLNANCDLKICDFGLARVTSETDFMTEYV<br>VTRWYRAPELLLNSADYTAAIDVWSVGCIFMELMDRRPLFPGRD<br>HVHQLRLLMELIGTPSEAELGFLNENAKRYIRQLPLYRRQSFTEKF<br>PHVHPAAIDLVEKMLTFDPRLRITVEDALAHPYLTSLHDISDEPVC<br>MTPFSFDFEQHALTEEQMKELIYREALAFNPEY
NP_001235426.1<br>[Glycine max]	MEGGGAAPPADTVMSDAAPPPQQAMAMGIENIPATLSHGGRFIQY<br>NIFGNIFEVTAKYKPPIMPIGKGAYGIVCSALNSETNEHVAIKKIAN<br>AFDNKIDAKRTLREIKLLRHMDHENVVAIRDIVPPPQREIFNDVYIA<br>YELMDTDLHQIIRSNQGLSEEHCQYFLYQILRGLKYIHSANVLHR<br>DLKPSNLLLNANCDLKICDFGLARVTSETDFMTEYVVTRWYRAP<br>ELLLNSSDYTAAIDVWSVGCIFMELMDRKPLFPGRDHVHQLRLL<br>MELIGTPSEADLGFLNENAKRYIRQLPLYRRQSFQEKFPHVHPEAI<br>DLVEKMLTFDPRKRITVEDALAHPYLTSLHDISDEPVCMTPFNFDF<br>EQHALTEEQMKELIYREALAFNPEYQ
XP_028205744.1<br>[Glycine soja]	MEGGGAAPPADTVMSDAAPPPQQAMAMGIENIPATLSHGGRFIQY<br>NIFGNIFEVTAKYKPPIMPIGKGAYGIVCSALNSETNEHVAIKKIAN<br>AFDNKIDAKRTLREIKLLRHMDHENVVAIRDIVPPPQREIFNDVYIA<br>YELMDTDLHQIIRSNQGLSEEHCQYFLYQILRGLKYIHSANVLHR<br>DLKPSNLLLNANCDLKICDFGLARVTSETDFMTEYVVTRWYRAP<br>ELLLNSSDYTAAIDVWSVGCIFMELMDRKPLFPGRDHVHQLRLL<br>MELIGTPSEADLGFLNENAKRYIRQLPLYRCQSFQEKFPHVHPEAI<br>DLVEKMLTFDPRKRITVEDALAHPYLTSLHDISDEPVCMTPFNFDF

	EQHALTEEQMKELIYREALAFNPEYQ
XP_017409341.1 [Vigna angularis]	MEGGGGAAPPADTVMSDAAPHPEPHQPPTAMGIDNIPATLSHGGK FIQYNIFGNIFEVTAKYKPPIMPIGKGAYGIVCSALNSETNEHVAIK KIANAFDNKIDAKRTLREIKLLRHMDHENVVAIRDIVPPPQREIFN DVYIAYELMDTDLHQIIRSNQALSEEHCQYFLYQILRGLKYIHSAN VLHRDLKPSNLLLNANCDLKICDFGLARVTSETDFMTEYVVTRW YRAPELLLNSSDYTAAIDVWSVGCIFMELMDRKPLFPGRDHVHQ LRLLMELIGTPSEADLGFLNENAKRYIRQLPPYHRQSFQEKFPHVH PEAIDLVEKMLTFDPRKRITVEDALAHPYLTSLHDISDEPVCMTPFS FDFEQHALTEEQMKELIYREALAFNPEYQ
AID46462.1 [Vigna mungo]	MEGGGGAAPPADTVMSDAAPHPEPHQPPAAAMGIDNIPATLSHG GRFIQYNIFGNIFEVTAKYKPPIMPIGKGAYGIVCSALNSETNEHVA IKKIANAFDNKIDAKRTLREIKLLRHMDHENVVAIRDIVPPPQREIF NDVYIAYELMDTDLHQIIRSNQALSEEHCQYFLYQILRGLKYIHSA NVLHRDLKPSNLLLNANCDLKICDFGLARVTSETDFMTEYVVTR WYRAPELLLNSSDYTAAIDVWSVGCIFMELMDRKPLFPGRDHVH QLRLLMELIGTPSEDDLGFLNENAKRYIRQLPPYLRQSFQEKFPHV HPEAIDLVEKMLTFDPRKRITVEDALAHPYLTSLHDISDEPVCMTP FSFDFEQHALTEEQMKELIYREALAFNPEYQ
XP_014509555.1 [Vigna radiata var. radiata]	MEGGGGAAPPADTVMSDAAPHPEPHQPPAAAMGIDNIPATLSHG GRFIQYNIFGNIFEVTAKYKPPIMPIGKGAYGIVCSALNSDTNEHVA IKKIANAFDNKIDAKRTLREIKLLRHMDHENVVAIRDIVPPPQREIF NDVYIAYELMDTDLHQIIRSNQALSEEHCQYFLYQILRGLKYIHSA NVLHRDLKPSNLLLNANCDLKICDFGLARVTSETDFMTEYVVTR WYRAPELLLNSSDYTAAIDVWSVGCIFMELMDRKPLFPGRDHVH QLRLLMELIGTPSEDDLGFLNENAKRYIRQLPPYLRQSFQEKFPHV HPEAIDLVEKMLTFDPRKRITVEDALAHPYLTSLHDISDEPVCMTP FSFDFEQHALTEEQMKELIYREALAFNPEYQ
NP_181907.1 [Arabidopsis thaliana]	MDGGSGQPAADTEMTEAPGGFPAAAPSPQMPGIENIPATLSHGGR FIQYNIFGNIFEVTAKYKPPIMPIGKGAYGIVCSAMNSETNESVAIK KIANAFDNKIDAKRTLREIKLLRHMDHENIVAIRDIIPPPLRNAFND VYIAYELMDTDLHQIIRSNQALSEEHCQYFLYQILRGLKYIHSANV LHRDLKPSNLLLNANCDLKICDFGLARVTSESDFMTEYVVTRWY RAPELLLNSSDYTAAIDVWSVGCIFMELMDRKPLFPGRDHVHQLR LLMELIGTPSEEELEFLNENAKRYIRQLPPYPRQSITDKFPTVHPLAI DLIEKMLTFDPRRRITVLDALAHPYLNSLHDISDEPECTIPFNFDFE NHALSEEQMKELIYREALAFNPEYQQ

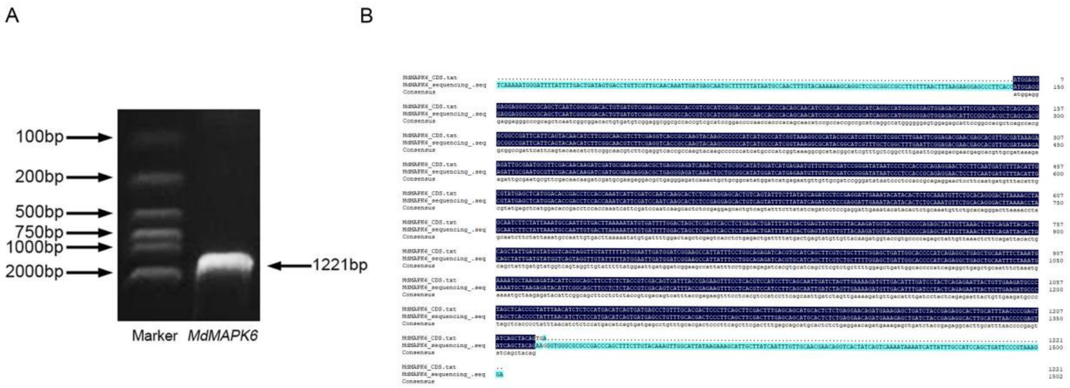

Supplementary Figure S1. Identification of the *MdMAPK6* gene. (A) DNA identification of the *MdMAPK6* gene. (B) DNA sequencing results of *MdMAPK6* gene.

```
        10         20         30         40         50         60         70
         |          |          |          |          |          |          |
MEGGGPAAQSADTVMSEAAPPSHPDPNHPQQHPPPPHQAMGGVESIPATLSHGGRFIQYNIFGNVFEVTA
ettcccchhhhheehtcccccccccccccccccccccccccccccceecttcceeeeeecccheeecc
KYKPPIMPIGKGAYGIVCSALNSETNEHVAIKKIANAFDNKIDAKRTLREIKLLRHMDHENVVAIRDIIP
ccccccccccccceeeeeehcctttcchheeehhhhhhhhhhhhhhhhhhhhhhhhhcctcceeeeeeeecc
PPQRNSFNDVYIAYELMDTDLHQIIRSNQALSEEHCQYFLYQILRGLKYIHSANVLHRDLKPSNLLLNAN
cccccccchheehhhhhhhhhhhhhhtccccchhhhhhhhhhhhhhhhhcchheehttccttceeeett
CDLKICDFGLARVTSETDFMTEYVVTRWYRAPELLLNSSDYTAAIDVWSVGCIFMELMDRKPLFPGRDHV
cceeeeeeeeccccccchhhhhhhhhhcccthheecccccchhhhhhhhhhhhhhhhhttcccccccchh
HQLRLLLELIGTPSEAELQFLNENAKRYIRQLPLYRRQSFTEKFPHVHPSAIDLVEKMLTFDPTQRITVE
hhhhhhhhhtcccccchhhhhhhhhhhhhhhccccccccchhhecccccchhhhhhhhhhecccttcceeeh
DALAHPYLTSLHDISDEPVCTTPFSFDFEQHALSEEQMKELIYREALAFNPEYQLQ
hhhhhhhhhhcccccccccccccccccccccchhhhhhhhhhhhhhhhctthccc

Sequence length :    406

SOPMA :
   Alpha helix      (Hh) :   170 is   41.87%
   3₁₀ helix        (Gg) :     0 is    0.00%
   Pi helix         (Ii) :     0 is    0.00%
   Beta bridge      (Bb) :     0 is    0.00%
   Extended strand  (Ee) :    53 is   13.05%
   Beta turn        (Tt) :    25 is    6.16%
   Bend region      (Ss) :     0 is    0.00%
   Random coil      (Cc) :   158 is   38.92%
   Ambiguous states (?)  :     0 is    0.00%
   Other states          :     0 is    0.00%
```

Supplementary Figure S2. Secondary structure of the *MdMAPK6* protein.

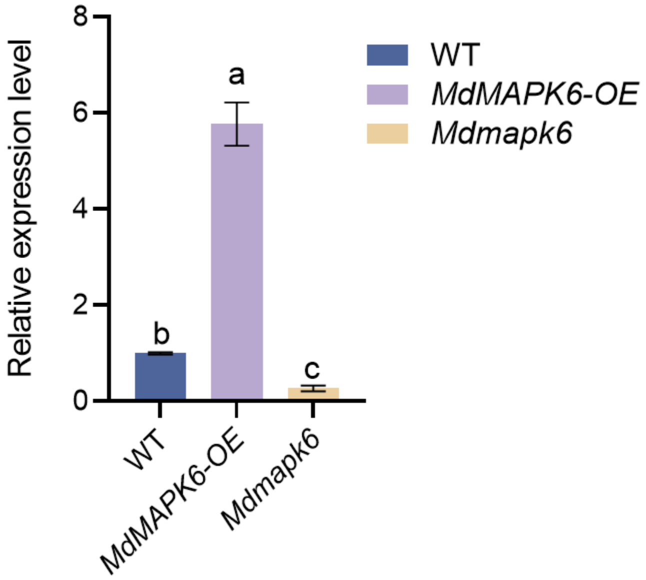

Supplementary Figure S3. Relative expression levels of MdMAPK6 in WT and MdMAPK6 transgenic lines detected by qRT-PCR.

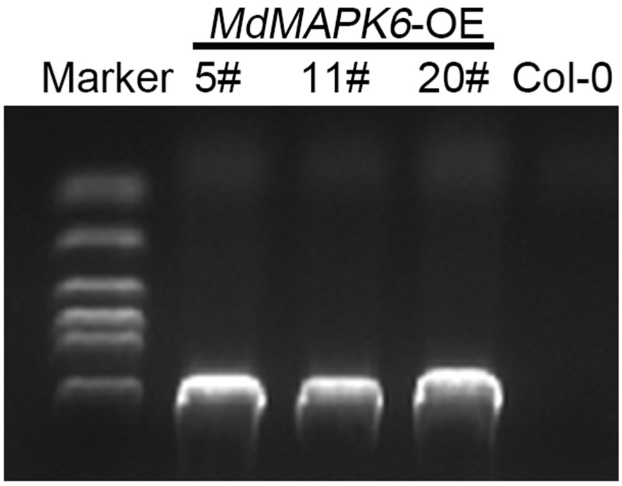

Supplementary Figure S4. DNA identification of *MdMAPK6* overexpressing transgenic lines of *A. thaliana*

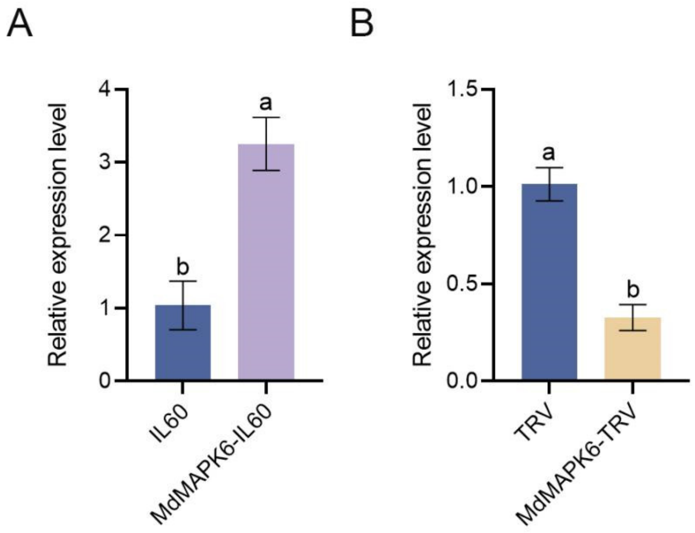

Supplementary Figure S5. The relative expression levels of *MdMAPK6* in apple fruit after three days of transient transformation. (A) The relative expression levels of *MdMAPK6* in silenced apple fruits. (B) The relative expression levels of *MdMAPK6* in overexpressing apple fruit. Data were obtained from three independent biological replicates. The different lower case letter markers represent significant differences and the significance level is taken as 0.05.
